# Reorganization of Mitochondrial Function and Architecture in Response to Plant‐Derived Alkaloids: Anatabine, Anabasine, and Nicotine, Investigated in SH‐SY5Y Cells and in a Cellular Model of Parkinson's Disease

**DOI:** 10.1111/cns.70571

**Published:** 2025-09-04

**Authors:** Dominika Malińska, Karolina Drabik, Bernadeta Michalska, Jarosław Walczak, Małgorzata Partyka, Monika Prill, Jędrzej Szymański, Paulina Patalas‐Krawczyk, Karolina Piecyk, Jerzy Duszyński, Mariusz R. Więckowski, Joanna Szczepanowska

**Affiliations:** ^1^ Nencki Institute of Experimental Biology Polish Academy of Sciences Warsaw Poland; ^2^ Institute of Fundamental Technical Research Polish Academy of Sciences Warsaw Poland

**Keywords:** anabasine, anatabine, mitochondria, mitochondrial remodeling, nicotine

## Abstract

**Aims:**

Nicotine, anatabine, and anabasine are the most prevalent alkaloids in *Nicotiana* species. While nicotine is the main addictive ingredient in tobacco products, it was also shown to have neuroprotective properties. Mitochondria appear to be one of the targets of nicotine in the cell. These multifunctional organelles are also the first responders to various cellular stresses. Thus, we characterized the impact of tobacco alkaloids on these organelles.

**Methods:**

We investigated the effects of structurally similar alkaloids, anatabine, anabasine, and nicotine, on mitochondrial function in SH‐SY5Y neuroblastoma cells under basal conditions and in the presence of rotenone, a mitochondrial stressor commonly used to model the cellular pathology underlying Parkinson's disease.

**Results:**

We observed changes in mitochondrial behavior, including hyperpolarization, alterations in mitochondrial network morphology, increased mitochondrial turnover rates, and upregulation of mitochondrial biogenesis regulators. The profiles of changes induced by particular alkaloids slightly differed; however, they shared many features with the stress response observed upon treatment with rotenone. Interestingly, the effects of the alkaloids and rotenone were not additive. Moreover, some parameters altered by rotenone were normalized upon cotreatment with the alkaloids.

**Conclusions:**

The results indicate that the investigated alkaloids stimulate mitochondrial stress adaptation. Despite structural similarity, they act through slightly different mechanisms.

## Graphical abstract

The effects of tobacco alkaloids: anatabine, anabasine, and nicotine, were investigated in a model of mitochondrial stress, mimicking cellular pathology accompanying Parkinson's disease development. The alkaloids modulated mitochondrial function and alleviated the hallmarks of mitochondrial stress. However, they acted via slightly different mechanisms.

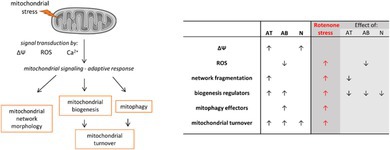

## 1 Introduction

Plant‐derived alkaloids are well‐known as important bioactive agents that have long received interest due to their therapeutic potential for chronic diseases such as diabetes, cancer, and neurological disorders [1]. There has been a strong focus on investigating alkaloids with simple chemical structures as templates for drug discovery. Moreover, owing to their abundance, alkaloids are potentially easy to extract and readily available for research. It is crucial to investigate and understand the action of these substances at the cellular and molecular levels before considering their medicinal use, as many alkaloids also have toxic effects. Thus, their safety profiles should be studied in detail.

We decided to examine the effects of the three most abundant tobacco alkaloids: nicotine, anatabine, and anabasine. Tobacco leaves contain more than 4000 alkaloids, with nicotine constituting 93%, anatabine 3.9%, and anabasine 0.5% of the alkaloid mixture present in fresh *Nicotiana tabacum* plants (the species most commonly used for cigarette tobacco production) [2]. Although tobacco is not considered a medicinal plant, some of its compounds may find therapeutic uses [3].

Nicotine is known for its role in tobacco addiction and as a pharmacological aid for smoking cessation [4]. However, its effects extend beyond these aspects. For example, the tobacco alkaloids nicotine and anatabine have shown anti‐inflammatory properties [5, 6, 7, 8]. In recent years, the neuromodulatory and neuroprotective actions of plant alkaloids have gained increasing attention from researchers. Among tobacco‐derived compounds, nicotine has been postulated to prevent the development of Parkinson's disease (PD) [9, 10]. However, the molecular mechanisms by which nicotine potentially affects the pathogenesis of PD remain unclear.

Nicotine, anatabine, and anabasine are agonists of nicotinic acetylcholine receptors (nAChRs) [11], and some of their biological activities, particularly within the nervous system, can be attributed to the activation of these receptors [12, 13]. However, nAChR‐independent activities have also been described for these alkaloids [5, 6, 14], with mitochondria suggested as one of their intracellular targets. Both beneficial and detrimental effects on mitochondrial function have been reported, such as attenuation of mitochondrial damage caused by mitochondrial permeability transition [15, 16], induction of mitochondrial oxidative stress, and alteration of mitochondrial dynamics [17].

Mitochondria are central organelles for cellular bioenergetics: they provide energy for basic metabolic processes, play key roles in regulating cellular metabolism, redox and calcium homeostasis, and act as signaling hubs in mammalian cells [18]. Consequently, compounds that modify their function can impact overall cellular physiology. Thus, it is important to understand the interactions of potential medicinal substances with mitochondria and cellular bioenergetics.

Mitochondria within the cell form networks that continuously adapt to fulfill their cellular functions [19]. The mitochondrial network is remodeled through changes in morphology, mitochondrial number, and distribution within the cell [20]. The reorganization of the mitochondrial network is inextricably linked to the processes of mitophagy and mitochondrial biogenesis, which regulate mitochondrial turnover and adaptation [21]. Under changing environmental demands, reprogrammed signaling in mitochondria plays a crucial role in maintaining metabolic flexibility and adjusting to bioenergetic demands [20].

Here, we studied the effects of the tobacco alkaloids anatabine, anabasine, and nicotine on mitochondrial function and organization. We assessed basic mitochondrial properties and functions in SH‐SY5Y neuroblastoma cells and found that these alkaloids affect mitochondrial network architecture, biogenesis, and turnover.

Furthermore, we extended our study to investigate the impact of these alkaloids on mitochondrial remodeling in a PD cellular model. SH‐SY5Y cells were treated with rotenone, a respiratory chain complex I inhibitor commonly used for modeling PD in vitro [22].

## 2 Materials and Methods

### 2.1 Reagents

All chemicals and reagents were of the highest analytical grade and purchased from Sigma Aldrich (Sigma Aldrich, Burlington, MA, USA), except when specified otherwise. We used (−)‐nicotine (Sigma Aldrich), anatabine citrate (synthesized by Concept Life Sciences, Manchester, UK), and (+)‐anabasine hydrochloride (Tocris Bioscience, Bristol, UK). The test compounds were prepared in dimethyl sulfoxide (DMSO) directly before each experiment. The antibodies were purchased from the following companies: against Tfam, beclin‐1, and p62 from Cell Signaling Technology (Danvers, MA, USA); against NRF1 and NRF2 from Proteintech (Rosemont, IL, USA); against Parkin and β‐actin (rabbit) from Abcam (Cambridge, UK); and against β‐actin (mouse) from Sigma–Aldrich.

### 2.2 Cell Line and Cell Culture

SH‐SY5Y neuroblastoma cells were obtained from the American Type Culture Collection (ATCC cat no CRL‐2266). The cells were cultured at 37°C in a humidified atmosphere of 5% CO_2_ in a 1:1 mixture of DMEM and F12 medium (Millipore, Burlington, MA, USA) supplemented with 10% fetal bovine serum (FBS) (Gibco, New York, NY, USA), 2 mM L‐glutamine (Gibco), 100 U/mL penicillin, and 100 μg/mL streptomycin. The cells were subcultured via trypsinization. For the experiments, the cells were seeded into appropriate culture vessels at a density of 40,000 cells/cm^2^ (for the proliferation and MTT assays) or 90,000 cells/cm^2^ (for the other assays). The treatments were started the next day after seeding.

### 2.3 Cell Cycle Analysis

The cell cycle was analyzed by flow cytometry upon staining of cellular DNA with propidium iodide (PI). The cells were seeded in 6‐well plates. After the treatments, the cells were detached via trypsinization and sedimented via centrifugation (5′, 200 × *g*). The pellet was washed twice with cold PBS and then suspended in 20 μL of PBS, and the suspension was rapidly added to an Eppendorf tube containing ice‐cold 70% ethanol. The samples were fixed for 24 h at −20°C, transferred to FACS tubes, and centrifuged for 5 min at 500 × *g*. The resulting pellets were washed two times with cold PBS and then suspended in 1 mL of a 1:1 mixture of PBS and extraction buffer (0.192 M Na_2_HPO_4_, 4 mM citric acid), incubated for 5 min at room temperature, and centrifuged for 5 min at 500 × *g*. The obtained pellets were suspended in 0.5 mL of PI staining solution (PBS with 3.8 mM sodium citrate, 50 μg/mL PI and 0.5 mg/mL RNAse A), incubated for 30 min in darkness, and then PI fluorescence was analyzed with a flow cytometer.

### 2.4 Cell Viability Assay

The cells were seeded in 96‐well plates. The next day after seeding, the treatment was started, and cell viability was determined after 1, 2, 3, and 4 days using 3‐(4,5‐dimethylthiazole‐2‐yl)‐2,5‐diphenyltetrazolium bromide (MTT) reduction assay.

### 2.5 Cell Proliferation Assay

The cells were seeded into 24‐well plates. Cell counts were performed using a Neubauer chamber on the day of starting the treatment (Day 0) and for 3 consecutive days. Trypan blue staining was performed to assess the number of viable cells. For calculation of the population doubling time, an exponential curve $y=ae^{\mathrm{bx}}$ was fitted to the obtained data of cell counts on consecutive days, and the population doubling time (PDT) was calculated as follows: $\mathrm{PDT}=\frac{\ln2}{b}$.

### 2.6 Measurements via Laser Scanning Cytometry

The cells were seeded on 24‐well plates. After treatment, the cells were loaded for 30 min with the appropriate fluorescent probe (see below), washed 2 times with fresh PBS, and then 0.5 mL of fresh PBS containing 0.9 M CaCl_2_, 0.49 M MgCl_2_ and 17.5 mM glucose was added to each well. The measurements were performed with an iCys laser scanning cytometer (Thorlabs, Newton, NJ, USA) with the use of a 20× objective. From each well, 12–16 fields of view were collected and analyzed. For probe loading, washing, and measurement, either culture medium or PBS supplemented with 0.9 M CaCl_2_, 0.49 M MgCl_2_, and 17.5 mM glucose was used. The following fluorescent probes were applied in particular assays:

– Cytosolic Ca^2+^ levels—4 μM fluo4‐AM fluorescent probe (*λ*_ex_ = 488 nm and *λ*_em_ = 530/30 nm);
– Cytosolic ROS levels—5 μM CM‐H_2_DCFDA (*λ*_ex_ = 488 nm and *λ*_em_ = 530/30 nm);
– Mitochondrial membrane potential (Δ*Ψ*) – 5 μM JC‐1 fluorescent probe (*λ*_ex_ = 488 nm, *λ*_em_ = 530/30 nm for JC1 monomers and 580/30 nm for JC1 aggregates);
– Mitochondrial mass—200 nM MitoTracker Green FM (*λ*_ex_ = 488 nm and *λ*_em_ = 530/30 nm).

The captured images were analyzed via iCys software version 2.5, as described previously [23]. The results were normalized to the mean values obtained in a particular experiment for the untreated controls.

### 2.7 Determination of Oxygen Consumption

SH‐SY5Y cells were seeded on culture plates (10 cm diameter), appropriately treated, and then detached by trypsinization, sedimented by centrifugation, and resuspended in 200 μL of culture medium devoid of FBS. Measurements were performed at 37°C with an Oroboros Oxygraph‐2 k (Oroboros, Innsbruck, Austria) in culture medium devoid of FBS. For each sample, the following parameters were measured: (i) basal respiration, (ii) oligomycin‐inhibited respiratory state 4 (in the presence of 0.1 μg/mL oligomycin), and (iii) CCCP‐uncoupled respiratory state III (protonophore CCCP titrated in 0.1 μM steps, the maximal oxygen consumption value obtained was used for further calculations).

### 2.8 Immunofluorescence Staining

The cells seeded on glass coverslips were stained with MitoRed (200 nM) for 20 min at 37°C and then briefly washed with culture medium. The cells were subsequently fixed with 4% paraformaldehyde in PBS (pH 7.4) for 15 min at room temperature, rinsed with PBS, and permeabilized with 5% Triton X‐100/PBS for 10 min. After three washes with 0.1% Triton X‐100/PBS, the cells were incubated in 0.1% Triton X‐100/PBS/2% BSA for 20 min. Subsequently, the anti‐α‐tubulin‐FITC antibody or phalloidin was diluted in the above buffer. The cells were incubated for 1 h and washed three times with 0.1% Triton X‐100/PBS. The nuclei were stained with Hoechst 33342, and the coverslips were mounted with Dako Glycergel on glass slides and visualized with a confocal microscope (Zeiss LSM780).

### 2.9 Cell Morphology and Mitochondrial Network Analysis

The cell morphology was described on the basis of confocal images of cytoskeleton staining using ImageJ software. Preprocessing included median filtering, contrast enhancement applying histogram normalization and Gaussian blur, upon which the contours of particular cells were extracted using Huang thresholding. The “Analyze particles” plugin was subsequently used to determine the following shape descriptors: area, circularity $\left(\frac{4\pi *\text{area}}{{\text{perimeter}}^{2}}\right)$, aspect ratio (the ratio of major to minor axes of the fitted ellipse), and solidity $\left(\frac{\text{area}}{\text{convex area}}\right)$.

For analysis of mitochondrial network morphology, the confocal image of MitoRed‐stained mitochondria was first preprocessed by median filtering and contrast enhancement using histogram normalization. The mitochondrial mask was subsequently obtained via Otsu thresholding, and the mitochondrial network morphology was characterized by determining the thickness of particular regions of the network and measuring the sizes of separated fragments of the network via the ImageJ plugins “Local Thickness” and “Analyze Particles”, respectively.

### 2.10 Visualization of Mitochondrial Age With the MitoTimer Vector

The cells were seeded in 24‐well plates. Twenty‐four hours after seeding, the cells were transfected with the MitoTimer vector using the TransfeX kit (ATCC, Manassas, VA, USA) in accordance with the manufacturer's recommendations. Twenty‐four hours after transfection, the cells were imaged via confocal microscopy (Zeiss Spinning Disc microscope) with an HC APO 63×/1.20 water objective. Quantification of MitoTimer fluorescence was performed with ImageJ software.

### 2.11 Whole‐Cell Extracts and Immunoblotting

Cells grown in 75 cm^2^ culture flasks were harvested and lysed in RIPA lysis buffer supplemented with protease and phosphatase inhibitor cocktails. The protein concentrations in the cell lysates were determined according to the Bradford method. The samples were supplemented with sodium dodecyl sulfate (SDS) sample buffer (0.5 M Tris–HCl, 2.3% SDS, 5% β‐mercaptoethanol [v/v], 12.5% glycerol [v/v], 0.025% bromophenol blue; pH 6.8). Lysates containing equal amounts of protein (15–20 μg) were separated using SDS–polyacrylamide gel electrophoresis, transferred to nitrocellulose or polyvinylidene difluoride membranes (Bio–Rad Laboratories, Hercules, CA, USA), blocked with Odyssey blocking buffer (LI‐COR Biosciences, Lincoln, NE, USA), and diluted 1:1 in Tris‐buffered saline for 1 h. The blots were incubated overnight with primary antibodies at the following dilutions: TFAM, 1:2000; Parkin, 1:1000; Beclin‐1, 1:1000; SQSTM1/p62, 1:1000; mouse β‐actin, 1:150000; rabbit β‐actin, 1:100000; NRF1, 1:1000; and NRF2, 1:1000. The blots were subsequently rinsed with PBS containing 0.1% Tween 20, incubated with fluorescently labeled secondary antibodies (LI‐COR Biosciences), and diluted 1:10,000 in Odyssey blocking buffer for 1 h. The fluorescent signal on the membrane was measured and quantified with the use of an Odyssey infrared imaging system (LI‐COR Biosciences). Full images of the membranes are available in the Supporting Information files (Data S1).

### 2.12 Statistical Analysis

All the experiments were performed in at least three independent repetitions. Normality of the distribution was checked with a Shapiro‐Wilk test. The data that exhibited normal distribution are expressed as the means ± standard deviations, and their statistical significance was assessed with Student's *t* test. The results of microscopic analyses at the single‐cell level, which did not meet the criteria of normality, are presented as medians and interquartile ranges (IQR), and were compared using the Mann–Whitney *U* test. In either case, *p* values < 0.05 were considered statistically significant.

## 3 Results

### 3.1 Determination of the Toxicity Thresholds of the Tested Substances in SH‐SY5Y Cells

**FIGURE 1:**
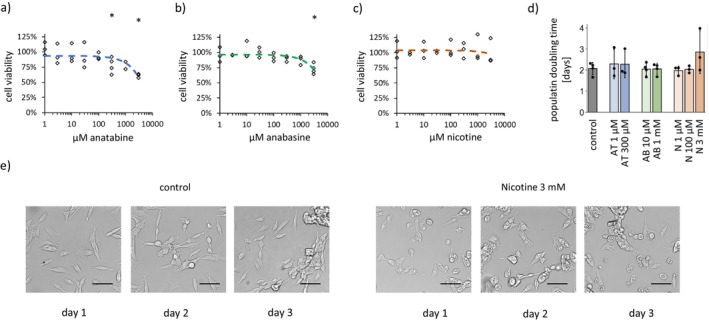
Determination of toxicity thresholds for the alkaloids anatabine, anabasine, and nicotine in SH‐SY5Y cells (a–c) Cell viability measured with the MTT assay after 2 days of treatment with (a) anatabine, (b) anabasine and (c) nicotine. (d) Population doubling times determined during 3 days of culture of SH‐SY5Y cells with the tested substances. The bar graphs present the means ± SDs from *n* = 3–4 independent repetitions. *p < 0.05 for Student's *t*‐test vs. untreated cells. AB, anabasine; AT, anatabine; N, nicotine. (e) Representative images of SH‐SY5Y cells on consecutive days of incubation with 3 mM nicotine compared with control cells. The scale bar indicates 50 μm.

Owing to the limited number of studies applying anatabine and anabasine in cell culture systems, we first determined the toxicity thresholds of these compounds in SH‐SY5Y cells. We investigated the effects of a wide range of concentrations (100 nM–3 mM) of the tested alkaloids on SH‐SY5Y cell viability, proliferation, and morphology (Figure 1 and Figure S1). For further tests, we aimed to select 2–3 concentrations of each of the alkaloids, including a concentration devoid of toxic effects and a dose close to the toxicity threshold.

The MTT cell viability test revealed that the toxic effects appeared only at very high, submillimolar or millimolar concentrations of the alkaloids: 300 μM anatabine or 1 mM anabasine (Figure 1a–c). A nicotine concentration of up to 3 mM did not affect the MTT test results. However, cell morphology was strongly altered by 3 mM nicotine (Figure 1e). 3 mM nicotine also significantly decreased SH‐SY5Y cell growth (Figure S1c and Figure 1d).

On the basis of these results, the following concentrations were selected for further studies: for anatabine, 1 μM (not toxic) and 300 μM (potential mild toxicity); for anabasine, 1 μM (not toxic) and 1 mM (potential mild toxicity); and for nicotine, 1 μM (not toxic), 100 μM (potential mild toxicity), and 3 mM (visible toxic effect). The treatment duration was set to 2 days.

### 3.2 The Impact of Anatabine, Anabasine and Nicotine on Hallmarks of Cellular Stress in SH‐SY5Y Cells

**FIGURE 2:**
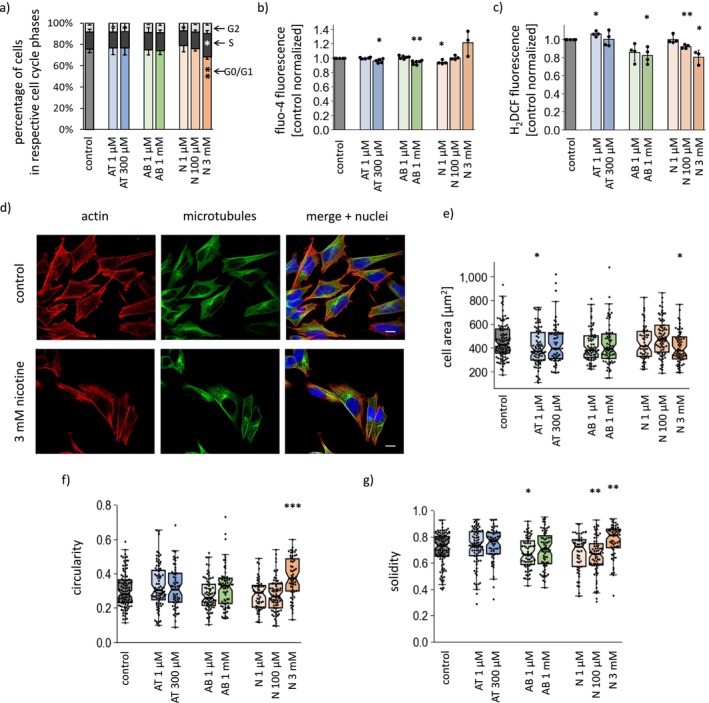
Impact of anatabine, anabasine and nicotine on the cell cycle profile, cellular ROS and Ca^2+^ homeostasis, and cell morphology. (a) The percentage of cells in particular stages of the cell cycle, (b) the levels of cytosolic Ca^2+^, and (c) the cytosolic ROS levels in different treatment options. The results of the Ca^2+^ and ROS measurements were normalized to the average values measured for the untreated cells. (d) Representative confocal images presenting cell morphology changes upon treatment with 3 mM nicotine. The cell nuclei (blue) were stained with DAPI, the Actin cytoskeleton (red) was stained with phalloidin‐Alexa 546, and the microtubules (green) were visualized with an anti‐α‐tubulin antibody. The scale bars indicate 10 μm. (e–g) Results of cell morphology analysis with shape descriptors: (e) cell area, (f) circularity and (g) solidity. The results are shown as the means ± SDs from *n* = 4–6 independent repetitions (a–c) or as median and IQR from analysis of 53–104 individual cell images obtained from 3 independent microscopy stainings (e–g). *p < 0.05, **p < 0.01, ***p < 0.001 vs. untreated cells. AB, anabasine; AT, anatabine; N, nicotine.

To determine whether the selected concentrations of nicotine, anatabine, and anabasine affect basic cellular homeostatic mechanisms and whether they can cause cellular stress in SH‐SY5Y cells, we examined the cell cycle profile, cell morphology, and cytosolic levels of Ca^2+^ and reactive oxygen species (ROS) (Figure 2 and Figure S2a).

All the measured parameters were affected by treatment with 3 mM nicotine. In line with the previously observed slower proliferation, the cell cycle profile revealed an accumulation of cells in S phase at the cost of a decline in the G0/G1 cell population (Figure 2a). The 3 mM nicotine also caused an increase in the cytosolic Ca^2+^ level (Figure 2b), while the ROS level was lower than that in the controls (Figure 2c). Examination of cell morphology revealed cytoplasmic vacuolization and visibly changed cell shape (Figure 2d), which was confirmed by analysis of shape descriptors (Figure 2e–g): cells treated with 3 mM nicotine were smaller than the control cells, more rounded (higher circularity) and had fewer protrusions (higher solidity).

The non‐toxic and sub‐toxic concentrations of the investigated alkaloids did not significantly affect the cell cycle or cell morphology. A slight decrease in cytosolic Ca^2+^ levels was detected in cells treated with higher doses of anatabine and anabasine, as well as with 1 μM nicotine, whereas 1 μM anatabine slightly increased cytosolic ROS. Interestingly, ROS levels were decreased by both anabasine and 100 μM nicotine.

### 3.3 The Effects of Anatabine, Anabasine, and Nicotine on Mitochondrial Network Morphology, Mitochondrial Function, and Turnover Rates in SH‐SY5Y Cells

**FIGURE 3:**
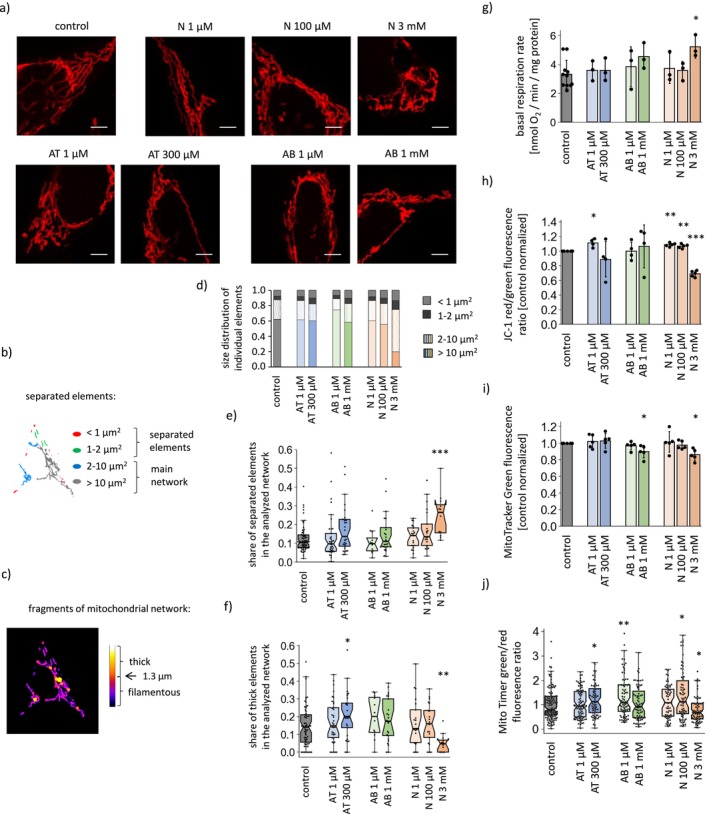
Impact of anatabine, anabasine and nicotine on mitochondrial structure and function (a) Representative confocal images of SH‐SY5Y cells mitochondria stained with MitoRed dye. The scale bar represents 5 μm. (b, c) Illustration of criteria for classifying the mitochondrial network fragments as separated elements (b) or as thick fragments (c). (d) Contribution of the elements of different sizes in the mitochondrial network in SH‐SY5Y cells under the applied treatments. (e) Share of separated elements (below 2 μm^2^) in the analyzed images of mitochondrial networks. (f) Share of thick fragments (above 1.3 um of width) in the mitochondrial networks of SH‐SY5Y cells under different treatment options. (g) Oxygen consumption rates, (h) mitochondrial membrane potential, (Δ*ψ*) (i) mitochondrial mass and (j) mitochondrial turnover rates in SH‐SY5Y cells treated with the tested alkaloids. The graphs present the means ± SDs from *n* = 4 (g, h) or *n* = 5 (i) independent experiments, or medians with IQR from the analysis of 12–96 (e, f) or 60–120 (j) individual cell images obtained in 3 independent microscopic stainings. *p < 0.05, **p < 0.01, ***p < 0.001 compared to untreated cells. AB, anabasine; AT, anatabine; N, nicotine.

Cytosolic Ca^2+^ and ROS levels are both sensitive to changes in mitochondrial function. We performed a more detailed investigation of mitochondrial physiology, including analysis of respiration rates, mitochondrial membrane potential (Δ*Ψ*), the morphology of the mitochondrial network, and mitochondrial mass and turnover rates (Figure 3).

The morphology of the mitochondrial network was strongly affected by 3 mM nicotine (Figure 3a–f): the mitochondria were thinner (thick fragments accounted for 5% of the network, whereas in untreated cells, it was 15%); and the network was more fragmented (small elements separated from the main network accounted for 25% of the mitochondrial content, while those separated from the main network accounted for 12% of the control). Milder changes were detected in the case of 300 μM anatabine: a slightly increased contribution of separated elements (to 18%) and thicker parts of the mitochondrial network (to 21%). Similar trends were observed for 1 mM anabasine and 100 μM nicotine.

The mitotoxic effect of 3 mM nicotine was visible in a strong decrease in the Δ*Ψ*, accompanied by a nearly twofold increase in the basal and uncoupled respiration rates (Figure 3g,h and Figure S3a–c), indicating that the detrimental effects on mitochondrial function were compensated to a certain extent by increased respiratory chain activity. Non‐toxic and sub‐toxic doses of anatabine, anabasine, and nicotine had no effect on cellular respiratory parameters. However, increased Δ*Ψ* was detected in cells treated with 1 μM anatabine and 1 and 100 μM nicotine, which indicates rearrangements in mitochondrial function.

The mitochondrial mass decreased upon treatment with 1 mM anabasine and 3 mM nicotine (Figure 3i). Additionally, 3 mM nicotine increased the average mitochondrial age (Figure 3j). In contrast, mitochondrial renewal was faster in SH‐SY5Y cells treated with 300 μM anatabine, 1 μM anabasine, and 100 μM nicotine.

### 3.4 The Levels of Proteins Involved in Mitochondrial Biogenesis and Mitophagy in SH‐SY5Y Cells Treated With Anatabine, Anabasine, and Nicotine

**FIGURE 4:**
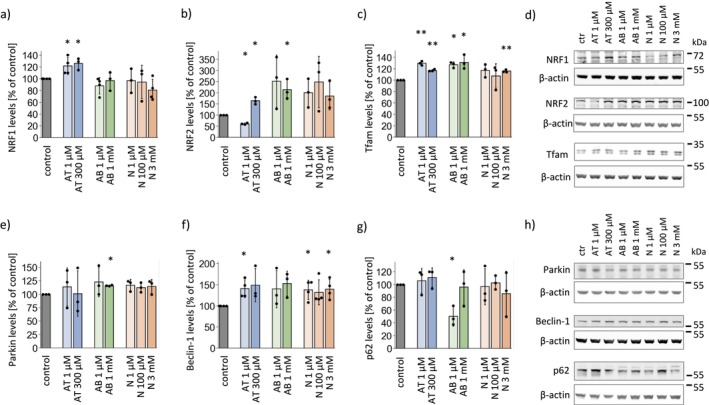
Levels of proteins regulating mitochondrial biogenesis and auto/mitophagy in SH‐SY5Y cells treated with anatabine, anabasine, and nicotine. The levels of (a) NRF1, (b) NRF2, and (c) Tfam in SH‐SY5Y cells treated with the investigated alkaloids. (d) Representative Western blots showing the levels of mitochondrial biogenesis regulators. The protein levels of (e) Parkin, (f) Beclin‐1 and (g) p62 in SH‐SY5Y cells upon treatment with the investigated alkaloids. (h) Representative Western blots showing the levels of proteins involved in auto/mitophagy. The graphs present the means ± SDs from *n* = 3–4 independent cell lysate sets. *p < 0.05, **p < 0.01, ***p < 0.001 vs. untreated cells. AB, anabasine; AT, anatabine; N, nicotine.

Mitochondrial age is determined by mitochondrial turnover rates, which in turn depend on the intensity of mitochondrial biogenesis and mitophagy. Western blot analysis revealed heterogeneous effects of the applied treatments on the levels of proteins regulating mitochondrial biogenesis and auto/mitophagy (Figure 4).

Anatabine significantly increased the levels of transcription factors stimulating mitochondrial biogenesis: NRF1 and Tfam. Interestingly, it had divergent effects on NRF2 levels, which, apart from mitochondrial biogenesis, is also involved in the regulation of antioxidant defense: 1 μM anatabine decreased, whereas 300 μM anatabine increased NRF2 levels. With respect to the proteins that mediate auto‐ and mitophagy, anatabine did not affect the levels of most of them, with the exception of beclin‐1, which was upregulated by approx. 40% by all of the tested substances.

Similarly to anatabine, anabasine increased the levels of Tfam; it also significantly increased the levels of NRF2. The analysis of auto and mitophagy markers suggested stimulation of this process by anabasine, as there was an increase in the protein levels of Parkin and beclin‐1, whereas p62 levels were significantly decreased by 1 μM anabasine. p62 is degraded during the course of autophagy; thus, its levels are commonly used as a marker of autophagy stimulation.

Nicotine caused a statistically significant increase in the levels of Tfam and beclin‐1. A trend toward NRF2 elevation was also observed.

### 3.5 Impact of Anatabine, Anabasine, and Nicotine on Mitochondrial Function in SH‐SY5Y Cells Challenged With Mitochondrial Stress Caused by Rotenone Treatment

**FIGURE 5:**
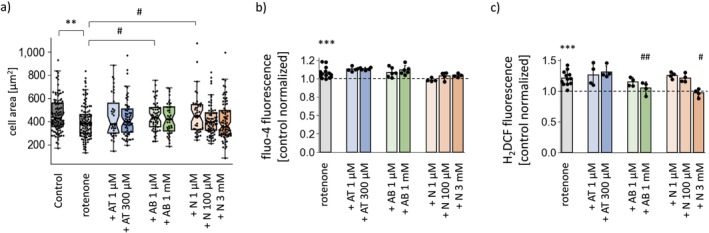
Effects of anatabine, anabasine, and nicotine on hallmarks of cellular stress induced by rotenone treatment in SH‐SY5Y cells. (a) Quantification of the cell area on the basis of confocal images. (b) Levels of cytosolic Ca^2+^. (c) Cytosolic ROS levels. The results of the Ca^2+^ and ROS measurements were normalized to the average values measured for the untreated cells. The results are shown as the means ± SDs from *n* = 4–6 independent experiments (b, c) or as medians with IQR from analysis of 34–116 individual cell images obtained from 3 independent microscopic stainings (a). The dashed lines represent the average values for the untreated controls. *p < 0.05, **p < 0.01, ***p < 0.001 comparing rotenone‐treated cells with controls; ^#^ *p* < 0.05, ^##^ *p* < 0.01 vs. rotenone‐treated cells. AB, anabasine; AT, anatabine; N, nicotine.

**FIGURE 6:**
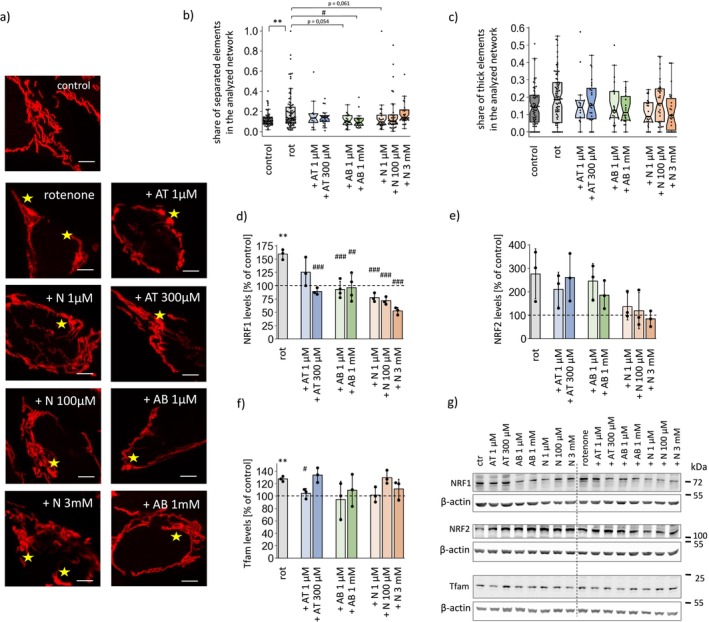
Effects of anatabine, anabasine and nicotine on hallmarks of cellular stress induced by rotenone treatment in SH‐SY5Y cells. (a) Representative confocal images of mitochondrial morphology in SH‐SY5Y cells treated for 2 days with 100 nM rotenone alone or in combination with the investigated alkaloids. The “clumpy” mitochondrial network fragments are marked with stars. (b) Contribution of separated elements and (c) share of thick fragments in the mitochondrial network in SH‐SY5Y cells under the applied treatments. The analysis was performed as described in the legend to Figure 3. (d–f) The levels of the mitochondrial biogenesis regulators NRF1 (d), NRF2 (e) and Tfam (f) in SH‐SY5Y cells upon the applied treatments and (g) representative Western blot results. The plots present the means ± SDs from the *n* = 3–4 independent lysate sets (d–f) or medians with IQR from the analysis of *n* = 13–94 individual cells from 3 independent microscopic stainings (b, c). The dashed lines mark the averages measured for the untreated controls. *p < 0.05, **p < 0.01, ***p < 0.001 when comparing rotenone‐treated cells with controls; ^#^ *p* < 0.05, ^##^ *p* < 0.01, ^###^ *p* < 0.001 vs. rotenone‐treated cells. AB, anabasine; AT, anatabine; N, nicotine.

We also investigated the effects of the alkaloids on cellular and mitochondrial function under conditions of mitochondrial stress caused by rotenone, a respiratory complex I inhibitor. Rotenone treatment is commonly used to model the cellular events underlying Parkinson's disease development [24]. To account for the characteristics of a chronic disease, we optimized the concentration of rotenone to cause cellular stress in the SH‐SY5Y cell line without inducing immediate cell death (Figure S4). The applied tests revealed that during 2 days of treatment, rotenone concentrations up to 100 nM did not lead to massive cell detachment and did not increase cell mortality—contrary to higher doses (200 nM) or longer treatment times (3 days). The 50 and 100 nM concentrations were selected for the subsequent experiments. Changes in cellular homeostasis caused by rotenone treatment manifested as altered cellular morphology (Figure 5a; Figures S2b and S5a,b) and increased cytosolic Ca^2+^ and ROS levels (Figure 5b,c). Rotenone treatment resulted in a twofold decrease in respiration rates; however, the Δ*Ψ* was maintained (Figure S5c–f), confirming the mild character of mitochondrial stress. We also observed small but consistent changes in mitochondrial network morphology (Figure 6a–c), with a greater prevalence of thick fragments and a greater contribution of the elements separated from the network. Additionally, rotenone‐induced mitochondrial stress caused increased mitochondrial turnover, along with increased levels of proteins that regulate mitochondrial biogenesis and are involved in mitophagy and autophagy (Figure 6d–g and Figure S5g–i).

Coadministration of anatabine, anabasine, or nicotine with rotenone did not affect SH‐SY5Y cell viability (data not shown). However, the morphology of the rotenone‐treated cells was slightly improved by 1 μM anabasine and 1 μM nicotine (Figure 5a and Figure S2b), whereas 1 mM anabasine decreased the cytosolic ROS levels in the rotenone‐treated cells (Figure 5c), similar to what was previously observed in the control cells.

Neither of the tested compounds was able to limit rotenone‐induced respiratory decline (Figure S5d–f). In contrast, some of the alkaloids reversed the changes in mitochondrial network morphology caused by rotenone: 1 mM anabasine and 1 μM nicotine tended to decrease the prevalence of thicker, “clumpy” fragments in the mitochondrial network in rotenone‐treated SH‐SY5Y cells (Figure 6c), and limited rotenone‐induced mitochondrial network fragmentation (Figure 6b).

Rotenone‐induced mitochondrial stress strongly affected the levels of proteins that mediate both biogenesis (increases in Tfam and NRF1 levels, Figure 6d–g) and auto/mitophagy (increases in beclin‐1 and Parkin levels, Figure S5g–i). Interestingly, the effects of rotenone were not additive with the previously mentioned effects of anatabine, anabasine, and nicotine, as the rotenone‐induced increase in NRF1 levels was abolished by nearly all of the applied alkaloid treatments, including 300 μM anatabine, which, when administered alone, increased NRF1 levels. The increase in Tfam caused by rotenone was reversed by coadministration of 1 μM anatabine, anabasine, or nicotine, which had opposite effects when administered alone. In contrast, cotreatment with alkaloids had no impact on the rotenone‐induced increase in the levels of auto‐, and mitophagy proteins, but their stimulatory effects on beclin‐1 and Parkin levels were not additive with the rotenone effect.

## 4 Discussion

Mitochondria play a central role in cellular energy metabolism, and their proper function is essential for cell survival, particularly in cell types with high energy demands, such as neuronal cells. The impaired regulation of mitochondrial function can disrupt cellular homeostasis and can contribute to many disorders. Conversely, mitochondria are common therapeutic targets in numerous diseases. These organelles act as signaling hubs in the regulation of cellular metabolism; they also dynamically respond to cellular stresses and adapt their function to allow cell survival under unfavorable conditions. Important elements of mitochondrial adaptation are adjustment of the mitochondrial network architecture (through fusion and fission) and mitochondrial turnover rates (through mitochondrial biogenesis and mitophagy). Therefore, we investigated these processes very carefully.

Therapies based on natural substances derived from plants have always symbolized the extraordinary phenomenon of symbiosis in the body and hold promise for treating various diseases. A wide array of natural substances is used; however, they are not free of limitations concerning their potency, side effects, and intolerability. They are usually not investigated at the molecular or cellular level. Moreover, mixtures of substances derived from tobacco have been more frequently studied, whereas the individual contributions of each alkaloid have not been evaluated [25, 26].

Alkaloids display a broad spectrum of molecular targets and have antioxidant and anti‐inflammatory properties. They are especially known as components of chemotherapy for treating cancer and neurodegenerative diseases [27, 28]. We chose three dominant alkaloids from tobacco: small‐molecule chemicals composed of pyridine and pyrrolidine rings, with similar chemical structures: nicotine, anatabine, and anabasine. Many studies have investigated the action of nicotine at the cellular level because of its presence in cigarettes and its potential effects on Parkinson's disease. Unfortunately, there is a lack of similar research on anabasine or anatabine.

1. We investigated a wide range of concentrations of substances because their effects on the cell depend on the concentration and can have either toxic or supportive effects on mitochondrial adaptation. We were particularly interested in the effects of low concentrations of these substances.
2. In this study, we applied two types of experimental conditions: neuroblastoma cells under standard culture conditions and cells subjected to stress induced by inhibition of complex I of the respiratory chain with rotenone, a widely recognized and thoroughly researched PD model.

### 4.1 Mechanism of Action of Anatabine

Anatabine is known as a dietary supplement effective for treating joint pain, stiffness, and functionality [8, 29, 30]. It has been shown to have anti‐inflammatory and neuroprotective properties [7, 8, 14]. Most studies on the effects of anatabine have been conducted in animal models; but studies on its effects at the molecular level on cell bioenergetics are lacking.

We chose two concentrations of this alkaloid for testing: one that does not affect the basic properties of SH‐SY5Y cells (referred to as “not toxic”, 1 μM) and a concentration that has a mild effect on cellular growth (referred to as “close to the toxicity threshold”, 300 μM).

Anatabine, even at a concentration of 1 μM, caused an increase in the mitochondrial membrane potential (Δ*Ψ*), indicating that mitochondria are among its intracellular targets. We also observed an increase in ROS levels, which may be related to its impact on mitochondria, as these organelles are among the main ROS producers in cells, and mitochondrial hyperpolarization leads to increased mitochondrial ROS synthesis [31]. Both the Δ*Ψ* and ROS levels trigger retrograde signaling from the mitochondria to the nucleus, activating factors that remodel mitochondrial function. Increased levels of NRF1 and Tfam, important regulators of mitochondrial biogenesis, show that anatabine treatment triggered a mitochondrial retrograde signaling response. NRF1 regulates the expression of many nuclear genes required for mitochondrial respiratory function, whereas Tfam is vital for mtDNA transcription and the expression of mitochondrial genes [32].

In the case of 300 μM anatabine, in addition to increased biogenesis regulators, we also detected greater mitochondrial turnover (lower mitochondrial age). In contrast, we observed no significant changes in the levels of auto/mitophagy effectors. However, notably, mitophagy can be executed by several different mechanisms and pathways [33]. At 300 μM, anatabine also affected mitochondrial network organization by increasing network fragmentation, which is potentially a factor facilitating mitochondrial turnover and renewal [34].

Other studies have shown that in SH‐SY5Y cells, anatabine at a concentration of 400 μM activates NRF2, the transcription factor that regulates the expression of some respiratory chain subunits and antioxidant enzyme‐encoding genes [32]. In our study, we observed similar effects when 300 μM anatabine was used.

We have shown that anatabine, at both low and high concentrations, causes mitochondrial remodeling by activating biogenesis, altering the organization of the mitochondrial network, and increasing mitochondrial turnover (i.e., a greater proportion of newly formed mitochondria compared to older ones). At the current stage of research, it appears that anatabine, at the tested concentrations, triggers mitochondrial retrograde signaling, contributing to the maintenance of a healthy mitochondrial population within the cell.

Other pathways through which anatabine may contribute to mitochondrial adaptation should also be considered, as anatabine is widely recognized for its complex role in regulating cellular signaling pathways associated with inflammation. Anatabine has been shown to activate NRF2 (a transcription factor that induces the expression of mitochondrial and antioxidant enzyme genes), modulate MAPK signaling, and suppress pro‐inflammatory transcription factors. The role of mitochondrial function in inflammatory signaling is well established.

These findings suggest that anatabine could be a promising candidate for therapeutic exploration in conditions involving mitochondrial signaling and inflammation‐related remodeling. Further studies are needed to identify additional cellular pathways affected by anatabine‐induced changes in mitochondrial function.

### 4.2 Mechanism of Action of Anabasine

Anabasine is more toxic than nicotine and has neurotoxic effects at high doses [35], but is less toxic than anatabine, which was decreasing the viability of SH‐SY5Y cells at much lower concentrations. Studies of anabasine at the molecular level have shown that its primary intracellular targets are nAchR [36]. In our study, however, we limited ourselves to examining how it affects mitochondrial adaptation.

Anabasine decreased cell viability at a concentration of 3 mM (high dose). A lower concentration of 1 mM had no effect on cell proliferation, viability, or morphology, but it reduced cellular levels of Ca^2+^ and ROS, both of which are among the first mitochondrial signaling elements involved in the activation of retrograde signaling. Reduced levels of ROS and Ca^2+^ can affect mitochondrial mobility and dynamics. In SH‐SY5Y cells, we also observed changes in the morphology of the mitochondrial network. Intracellular calcium plays a regulatory role in mitochondrial dynamics by influencing their movement and spatial distribution within the cell (calcium modulates the activity of the key protein Miro, which governs mitochondrial transport along microtubules). A lower level of calcium may enhance mitochondrial transport within the cell, a process essential for proper energy distribution and intracellular signaling during cellular adaptation, especially when mitochondrial turnover is activated.

After 2 days of incubation, anabasine at both high and low concentrations induced functional and structural reorganization of mitochondria: the biogenesis process was activated, as shown by increases in the levels of NRF2 and Tfam, and in mitochondrial turnover. The reduced mass of mitochondria and increased level of Parkin indicate that mitophagy is also activated. Mitophagy and mitochondrial biogenesis are activated by anabasine, suggesting that the cell is working to restore mitochondrial homeostasis and maintain a healthy, functional mitochondrial population. This balance enables the selective removal of damaged mitochondria while simultaneously generating new ones, thereby supporting cellular energy demands, reducing oxidative stress, and preserving overall cell viability and function.

We propose that anabasine triggers the activation of retrograde signaling, leading to functional adaptation of mitochondria within the cell. An increase in both biogenesis and mitophagy may be critical for cell survival, particularly in neurons.

### 4.3 Mechanism of Action of Nicotine

Nicotine is known as a dangerous and highly addictive chemical. On the other hand, many scientific reports suggest that nicotine could be beneficial, as it has antiapoptotic or anti‐inflammatory properties [37]. Several studies have shown that nicotine has neuroprotective effects, acting through nAChRs and affecting different signaling pathways [37, 38]. Our data show that these pathways also include the mitochondrial stress response, leading to the reorganization of mitochondrial function.

The effects of different concentrations of nicotine have been studied in cellular and animal models. In our research, we tested three nicotine concentrations: 1 μM, which had no effect on cell viability or morphology; 100 μM, which had mild effects on cells but was still not toxic; and 3 mM, which was harmful to SH‐SY5Y cells (changed morphology and slowed proliferation). Our study revealed that the primary effect of nicotine on mitochondrial physiology involves an increase in the Δ*Ψ* at both very low (1 μM) and high (100 μM) concentrations. Maintaining sufficient Δ*Ψ* is essential for proper mitochondrial function, including ATP synthesis and metabolite transport. However, excessive hyperpolarization of the mitochondrial inner membrane is undesirable, as it may increase mitochondrial ROS production and lead to oxidative stress [31]. We did not observe ROS stimulation in nicotine‐treated cells. In contrast, 100 μM nicotine decreased the ROS levels. Similarly, others have shown that in isolated rat brain mitochondria, nicotine can lower ROS production by inhibiting complex I of the respiratory chain, thus reducing electron leakage [15, 39]. However, numerous studies on cultured cells (e.g., bronchial and lung epithelial cells, lung carcinoma cells, and mesangial cells) have shown that nicotine treatment increases cytosolic ROS and leads to oxidative stress, even at concentrations of approximately 1 μM [37]. For example, in cardiomyocytes, a low concentration of nicotine (100 nM, 48‐h incubation) leads to an increase in ROS levels. The authors suggest that cardiomyocyte damage may result from increased oxidative stress and mitochondrial dysfunction, as nicotine activates the ERK–4E‐BP1 signaling axis [40]. Mitochondrial involvement in the activation of the MAPK/ERK signaling cascade (triggered mainly by ROS, Ca^2+^, ATP levels, and other elements of the mitochondrial stress signaling pathway) is well known. Nicotine may activate distinct signaling pathways, mainly leading to elevated ROS production and the involvement of mitochondrial retrograde signaling. This, in turn, may cause mitochondria to adapt their function in response to altered cellular conditions. Another study shows that in SVG‐A fetal astrocytes, the ROS level increased after 30 min but was lower after longer incubation [41]. It is likely that 48 h of incubation with nicotine in SH‐SY5Y cells is sufficient to allow for mitochondrial adaptation. All of these studies suggest that the effect of nicotine may vary depending on the type of cells or tissue investigated, as well as the duration and concentration of exposure.

In our study, 100 μM nicotine increased the turnover of mitochondria while maintaining the balance between biogenesis and mitophagy. The increased population of young mitochondria, increased membrane potential, and diminished ROS levels indicate positive adaptive changes in the mitochondria of SH‐SY5Y cells. Godoy et al. [42] reported that in cultured hippocampal neurons incubated with 10 μM nicotine for 30–120 min, the organization of the mitochondrial network changes: shorter incubation times promote mitochondrial fission, whereas longer incubation promotes mitochondrial fusion. Additionally, mitochondrial biogenesis is activated by nicotine, whereas the Δ*Ψ* and mitophagy are not affected.

A very high concentration of nicotine (5 mM) induced mitochondrial fission in human periodontal ligament cells (hPDLCs), as nicotine was shown to promote the phosphorylation of Drp1 (a key protein involved in the fission process). Additionally, an increase in mitochondrial membrane potential, ATP production, and ROS levels was observed. The authors suggest that the effects of nicotine are mediated through the activation of the c‐Jun N‐terminal kinase (JNK) pathway [43]. All components of mitochondrial stress signaling—including mitochondrial fission, altered membrane potential, ATP levels, ROS production, and calcium fluxes—are closely linked to the activation of the JNK signaling pathway. Therefore, considering the importance of mitochondrial stress signaling in activating other cellular pathways in response to various stimuli is crucial.

Another study in which the human multipotent embryonal carcinoma cell line NT2/D1 was incubated for 24 h with 10 μM nicotine revealed that nicotine induces mitochondrial fragmentation, decreases intracellular ATP levels, and inhibits proliferation [44]. Borkar et al. [45] also studied the effect of 10 μM nicotine (48 h) on primary human airway smooth muscle cells and observed fragmentation of the mitochondrial network and disruption of oxidative phosphorylation. In spermatogonia stem cells, exposure to nicotine induced oxidative stress and mitochondrial hyper‐fusion and reduced mitophagy, ultimately leading to senescence [46]. Authors suggest that the sirtuins—SIRT6 pathway is possibly and partially involved in nicotine‐induced mitochondrial dynamic changes.

We observed a trend toward a more fragmented mitochondrial network with 100 μM nicotine. The effect of nicotine on mitochondrial function depends on the concentration, incubation time, and type (sensitivity) of the examined cells. The SH‐SY5Y cell line is a cancer line, which may make it more resistant to stress factors [47].

Clear toxic effects of nicotine were observed in SH‐SY5Y cells only at very high concentrations (3 mM). Similar results were obtained by Wang et al., who reported that 3 mM nicotine applied for 12 h to SH‐SY5Y cells induced mitochondrial defects, such as increased mtDNA damage and decreases in ROS levels, △*Ψ*m and mitochondrial mass [48]. However, it has also been demonstrated that high and very high doses of nicotine can elicit varying responses in different cellular models [49]. For example, nicotine at a concentration of 4 mM, following a 24‐h incubation, induces an upregulation of mitochondrial ROS. Notably, this increase in ROS contributes to autophagic flux. In this context, nicotine exerts a neuroprotective effect in neuronal cells, with the cellular response to this stress mediated through activation of the nicotinic acetylcholine receptor (nAChR) pathway (in this study, involvement of Trk proteins was shown as part of this mechanism). The authors suggest that nicotine exhibits a dual effect, promoting neurotoxic ROS production while simultaneously enhancing neurotrophin signaling pathways that support cellular survival.

Based on our current and previous findings, as well as those of other researchers, several conclusions can be drawn regarding the role of nicotine in mitochondrial function: (1) nicotine affects mitochondrial calcium and ROS regulation, which plays a critical role in numerous intracellular signaling pathways, (2) nicotine influences the mitochondrial membrane potential, thereby altering ATP synthesis and overall cellular energy metabolism, (3) nicotine can disrupt the delicate balance between mitochondrial fission and fusion processes, and (4) nicotine impacts mitochondrial biogenesis, mitophagy, and mitochondrial turnover.

Together, these findings underscore the multifaceted role of nicotine in both modulation of mitochondrial signaling and in related cellular pathways dependent on mitochondrial activity. However, the effects of nicotine on mitochondrial function are contingent upon its concentration, duration of exposure, and the specific cellular, tissue, or organismal model employed in the study.

### 4.4 Effects of Anatabine, Anabasine, and Nicotine on Neuroblastoma Cells Treated With Rotenone

Nicotine has been shown to have neuroprotective effects in models of neurodegenerative diseases [50, 51, 52]. In particular, its beneficial effects on PD have been studied and discussed. For example, nicotine significantly delayed mitochondrial swelling and cytochrome c release in a PD model in SH‐SY5Y cells [15]. Therefore, we selected SH‐SY5Y neuroblastoma cells incubated with rotenone as a model for our studies on the potential protective effects of tobacco alkaloids. Rotenone is an inhibitor of complex I of the respiratory chain, and complex I deficiency is a characteristic feature of PD [53, 54].

Since mitochondrial dysfunction is a key hallmark of PD, mimicked by rotenone treatment, we examined the posttreatment effects of anatabine, anabasine, and nicotine on mitochondrial behavior. Notably, this is an imperfect cellular model of PD, as the SH‐SY5Y cell line has tumorigenic properties that affect its differentiation fate, viability, growth efficiency, metabolic properties, and genome stability. Additionally, reports on the exact phenotype of SH‐SY5Y cells in the scientific literature are conflicting because of differences in cell origin and persistence in culture [24, 55].

We selected and used a concentration of rotenone that does not cause cell damage. In our experimental model, rotenone caused an increase in ROS, fragmentation of the mitochondrial network, activation of biogenesis and mitophagy, along with an increase in mitochondrial turnover, that is, features that occur in response to mitochondrial stress to promote cell survival. Uncontrolled biogenesis without quality control might lead to an increase in ROS if new mitochondria are not properly assembled. Biogenesis itself is an energy‐demanding process, which might stress neurons already struggling with ATP deficits.

**FIGURE 7:**
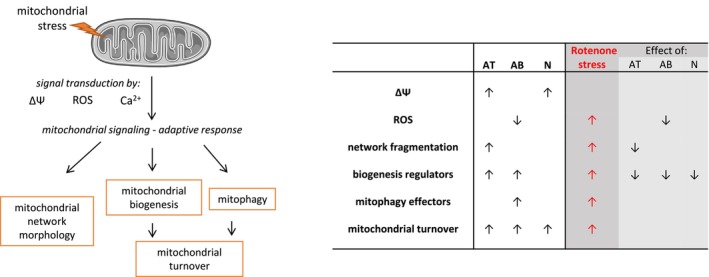
Schematic representation of the role of the investigated parameters in mitochondrial remodeling and summary of the observed effects of anatabine, anabasine and nicotine. The scheme depicts the interrelations between the measured parameters and their role in the mitochondrial stress response: The levels of ROS, Ca^2+^ and Δ*Ψ* act as indicators of mitochondrial condition and function. Changes in their levels trigger adaptive response, aimed at adjusting mitochondrial function by altering mitochondrial network morphology, biogenesis and mitochondrial removal, thus regulating mitochondrial mass and the renewal of the mitochondrial pool. In addition to the scheme, the observed effects of anatabine (AT), anabasine (AB), and nicotine (N) are summarized in the table next to the scheme.

We demonstrated that anatabine, anabasine, and nicotine diminished rotenone‐mediated toxicity. However, the targeted aspects of rotenone‐induced damage are different for each substance. Anatabine causes a change in the organization of mitochondria, restoring mitochondrial morphology (a less fragmented network). The level of the NRF1 transcription factor, which is responsible for regulating stress‐responsive gene expression, also decreases. In addition to improving mitochondrial morphology and normalizing NRF1 levels, anabasine also lowers ROS levels, whereas nicotine has the strongest effect on NRF1 and NRF2 levels (summary in Figure 7).

Each of the tested substances contributes to a reduction in mitochondrial biogenesis, which had been elevated due to the presence of the stressor rotenone. It is well known that mitochondria, under strong stress, must meet the increased energy demands of the cell. During such stress, there is an increased pool of mitochondria undergoing degradation, which in turn enhances both the biogenesis of new mitochondria and mitophagy to remove old, damaged, or dysfunctional ones.

It can be suggested that the substances studied help stabilize mitochondrial function and homeostasis. While mitochondrial biogenesis decreases, the level of mitophagy remains unchanged. It is possible that ongoing mitophagy is still sufficient to remove dysfunctional mitochondria, either because there is still a large pool of defective mitochondria in the cell or because the balance between mitophagy and biogenesis is dysregulated. As a result, the overall mitochondrial pool may appear stable despite the impaired generation of new mitochondria.

A comprehensive analysis was conducted on the interaction between nicotine and nicotinic acetylcholine receptors (nAChRs), highlighting their potential neuroprotective roles and associated molecular pathways [56]. Nourse and colleagues investigated whether nicotine activates nAChRs to selectively protect dopaminergic neurons in *C. elegans* [38]. The authors showed that nicotine protects dopaminergic neurons in the substantia nigra by modulating mitochondrial dynamics, particularly through the activation of mitochondrial quality control mechanisms.

The results showed that anatabine, anabasine, and nicotine cannot prevent mitochondrial respiratory dysfunction caused by rotenone, but the presence of these substances alleviated the symptoms of rotenone‐induced mitochondrial stress. More in‐depth molecular studies are needed to determine whether modulation of mitochondrial retrograde signaling by anatabine, anabasine, or nicotine could represent a viable therapeutic strategy for neurodegenerative disorders.

## 5 Brief Summary and Conclusions

In summary, the tobacco alkaloids anatabine, anabasine, and nicotine were not toxic to SH‐SY5Y cells up to sub‐millimolar concentrations. However, they influenced the cytosolic ROS and Ca^2+^ levels, indicating rearrangements within cellular homeostasis (Figure 7). Mitochondrial function was also affected by these compounds, as they induced changes in mitochondrial network morphology and the Δ*Ψ*. Additionally, mitochondrial turnover was stimulated in alkaloid‐treated SH‐SY5Y cells. The nature of all these effects was mild, suggesting an adaptive rather than detrimental character.

We emphasized that intracellular signaling leading to mitochondrial adaptation can occur via multiple pathways—not only through direct activation of mitochondrial retrograde signaling but also through nicotinic acetylcholine receptors (nAChRs) or the ERK signaling pathway. The profiles of the effects exerted by the individual alkaloids differed slightly, but they shared some common features, such as stimulated mitochondrial renewal and altered levels of mitochondrial biogenesis regulators. Moreover, the presence of alkaloids reversed some of the rotenone‐induced changes in mitochondrial network morphology, biogenesis regulation, and ROS levels.

## Author Contributions

**Dominika Malińska:** investigation, methodology, formal analysis, visualization, writing – original draft, writing – review and editing. **Karolina Drabik:** investigation, formal analysis. **Bernadeta Michalska:** investigation, formal analysis. **Jarosław Walczak:** investigation, formal analysis. **Małgorzata Partyka:** investigation, formal analysis. **Monika Prill:** investigation, formal analysis. **Jędrzej Szymański:** investigation, formal analysis. **Paulina Patalas‐Krawczyk:** investigation, formal analysis. **Karolina Piecyk:** investigation, formal analysis. **Jerzy Duszyński:** conceptualization, funding acquisition, writing – review and editing, supervision. **Mariusz R. Więckowski:** conceptualization, writing – review and editing. **Joanna Szczepanowska:** conceptualization, methodology, writing – original draft preparation, writing – review and editing, supervision.

## Conflicts of Interest

Philip Morris International provided financial support for the performance of the data generation. After completing the experimental part, the project was not continued. The following analysis, interpretation, and publication of the results are the sole responsibility of the authors.

## Supporting information


**Data S1:** cns70571‐sup‐0001‐DataS1.pdf.

**Data S2:** cns70571‐sup‐0002‐DataS2.docx.
**Figure S1:** (supplementary to Figure 1) Toxicity thresholds for the alkaloids anatabine, anabasine and nicotine in SH‐SY5Y cells. (a) Representative brightfield images of SH‐SY5Y cells treated for 1, 2, or 3 days with different concentrations of the tested alkaloids. The scale bar indicates 50 μm. (b) The results of cell viability determination with the MTT assay after 1, 2, and 3 days of treatment with different concentrations of the tested compounds. The data points present averages for Day 1 (diamonds), Day 2 (squares) and Day 3 (triangles) from 2 to 4 independent repetitions. (c) The impact of the tested compounds on cell counts after 3 days of treatment. The bar graphs present the means ± SDs from *n* = 3–4 independent repetitions. *p < 0.05 for Student's *t*‐test vs. untreated cells.
**Figure S2:** (supplementary to Figures 2 and 5) Impact of anatabine, anabasine and nicotine on the morphology of SHSY‐5Y cells. Representative confocal images of SH‐SY5Y cells treated for 2 days with the investigated alkaloids (a) alone or (b) in presence of 50 nM rotenone. The cell nuclei (blue) were stained with DAPI, the actin cytoskeleton (red) was stained with phalloidin‐Alexa 546, and the microtubules (green) were visualized with an anti‐α‐tubulin antibody. The scale bars indicate 10 μm. AB, anabasine; AT, anatabine; N, nicotine.
**Figure S3:** (supplementary to Figure 3) Analysis of the impact of anatabine, anabasine and nicotine on mitochondrial structure and function. (a) Representative measurement of oxygen consumption in SH‐SY5Y cells. (b) Impact of 2 days of treatment with the tested alkaloids on oxygen consumption in the presence of 1 μg/mL oligomycin and (c) maximal respiration obtained during CCCP titration. The graphs present the means ± SDs from *n* = 4 independent repetitions. *p < 0.05 for Student's *t*‐test vs. untreated cells. (d) Schematic representation of the image analysis workflow (detailed description in the Materials and Methods). (e) Overlaid images of green and red fluorescence in SH‐SY5Y cells transfected with the MitoTimer vector. Examples of cells with green/red fluorescence ratios corresponding to 270%, 90%, and 35% of the average ratio measured in untreated cells.
**Figure S4:** Determination of the rotenone concentration causing mild cellular stress in SH‐SY5Y cells. (a) The results of cell viability determination with the MTT assay after 1, 2 and 3 days of treatment with different rotenone concentrations. The data points present averages for Day 1 (diamonds), Day 2 (squares), and Day 3 (triangles) from 2 to 4 independent repetitions. (b) Cell viability was measured with an MTT assay after 2 days of treatment with selected rotenone concentrations. (c) Cell counts after 1, 2 and 3 days of treatment with selected rotenone concentrations. The results were normalized to the number of cells per well on the day of starting the treatment (Day 0). The black parts of the bars represent the number of trypan blue‐positive cells. (d) Representative images of SH‐SY5Y cells treated with selected rotenone concentrations for 1 and 2 days. The bar graphs in panel B present the means ± SDs from *n* = 6 and those in panel C from *n* = 3 independent experiments. *p < 0.05, **p < 0.01 according to Student's *t*‐test vs. untreated cells.
**Figure S5:** (supplementary Figures 5 and 6) Cellular morphology (a, b), mitochondrial function (c–f) and the levels of auto/mitophagy effectors (g–i) in SH‐SY5Y cells treated for 2 days with rotenone alone or in combination with the investigated alkaloids Dashed lines mark the average values measured in untreated cells. (a) Cell shape circularity, (b) cell shape solidity, (c) mitochondrial membrane potential, (d) basal respiration rates, (e) oligomycin‐inhibited respiration, (f) CCCP‐uncoupled respiration, (g) the levels of parkin, (h) beclin‐1 and (i) p62 proteins. Representative Western blots are shown below the graphs. The graphs present the means ± SDs from the analysis of *n* = 34–116 individual cells (a, b), *n* = 4 independent experimental repetitions or *n* = 3–4 independent lysate sets. *p < 0.05, ***p < 0.001 for Student's *t*‐test vs. untreated cells; ^#^ *p* < 0.05, ^##^ *p* < 0.01 for Student's *t*‐test vs. rotenone‐treated cells. AB, anabasine; AT, anatabine; N—nicotine.

## Data Availability

The data that support the findings of this study are available from the corresponding authors upon reasonable request.
