# Supplementary material for: Reorganization of Mitochondrial Function and Architecture in Response to Plant‐Derived Alkaloids: Anatabine, Anabasine, and Nicotine, Investigated in SH‐SY5Y Cells and in a Cellular Model of Parkinson's Disease
Source: CNS Neurosci Ther. 2025 Sep 4;31(9):e70571. doi: 10.1111/cns.70571 (PMC12409299; doi:10.1111/cns.70571)

Full unedited blots for figure 4 d

Channel 1 (800 nm)

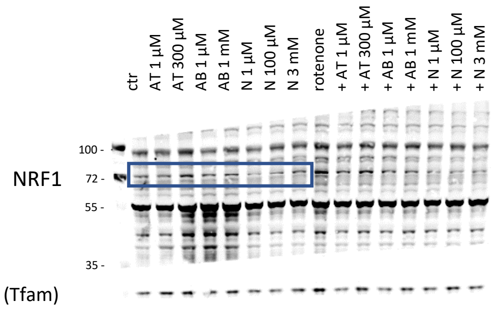

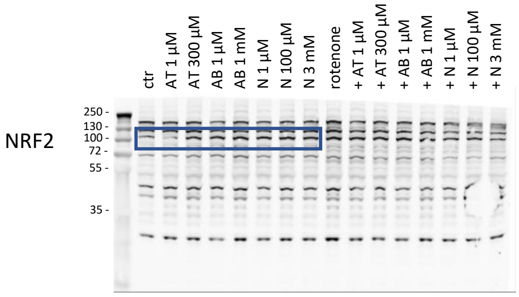

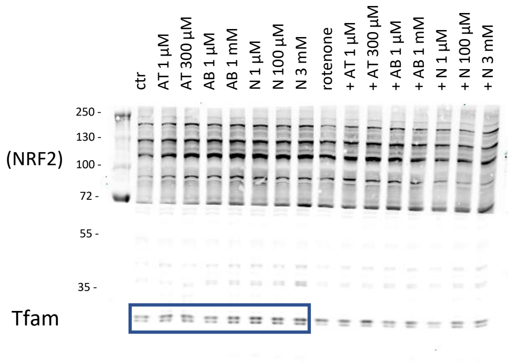

Channel 2 (680 nm)

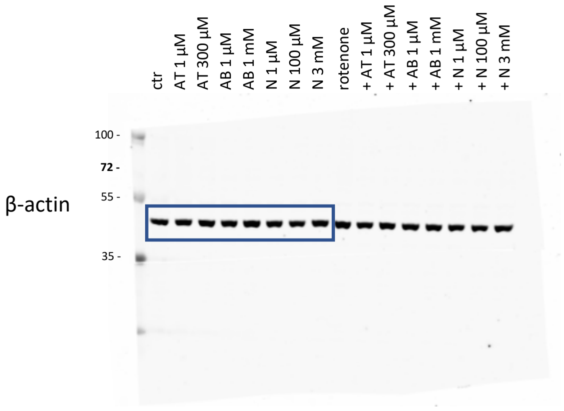

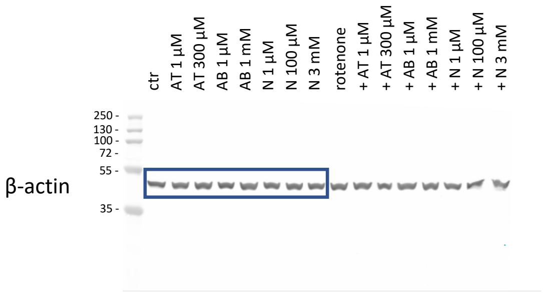

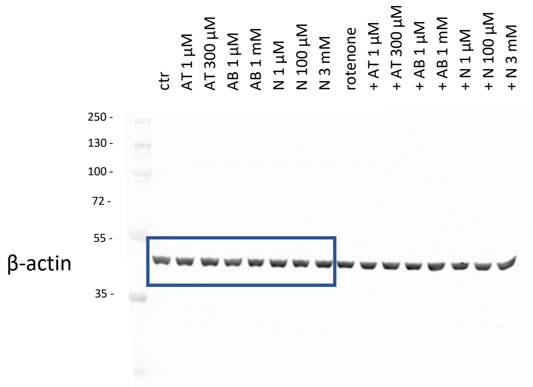

Full unedited blots for figure 4 h

Channel 1 (800 nm)

Channel 2 (680 nm)

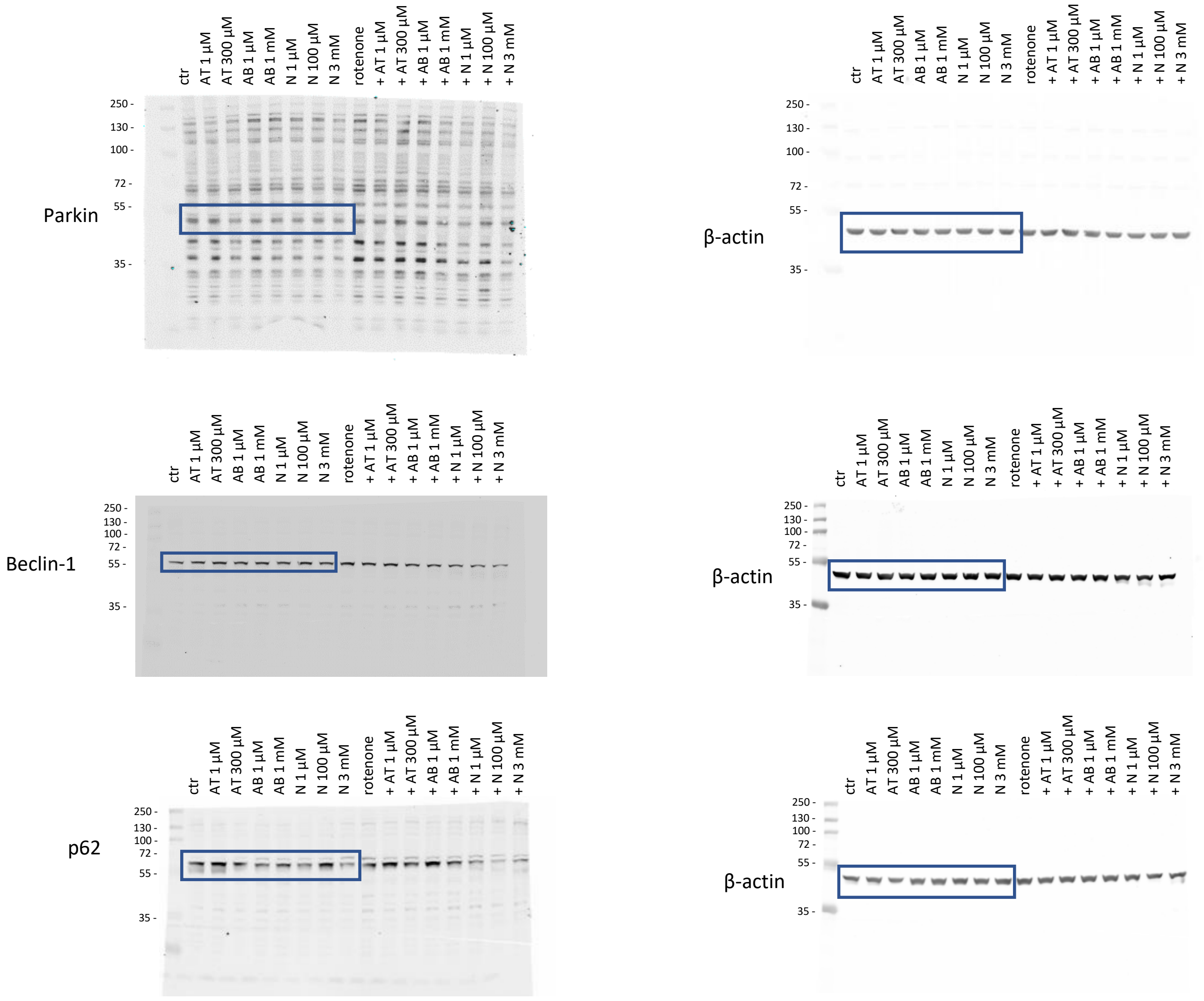

Full unedited blots for figure 6

Channel 1 (800 nm)

Channel 2 (680 nm)

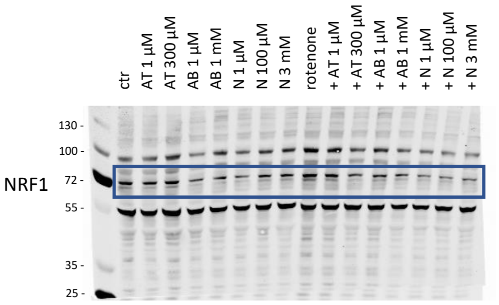

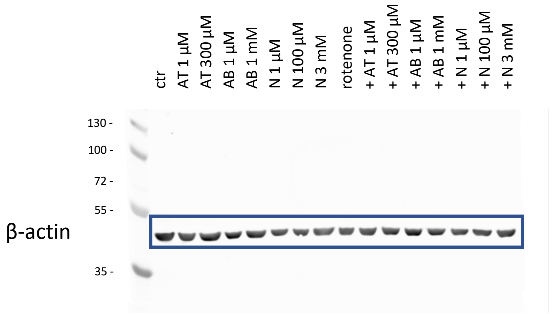

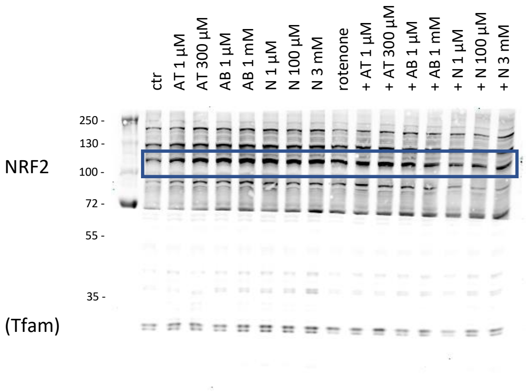

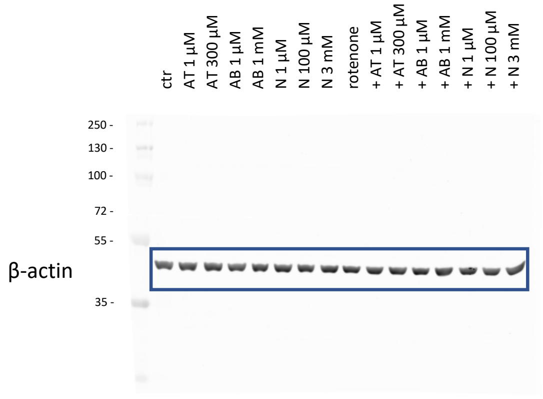

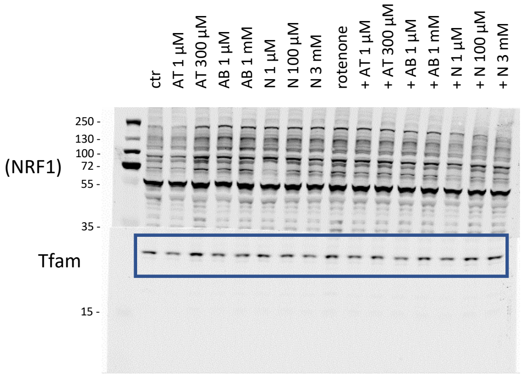

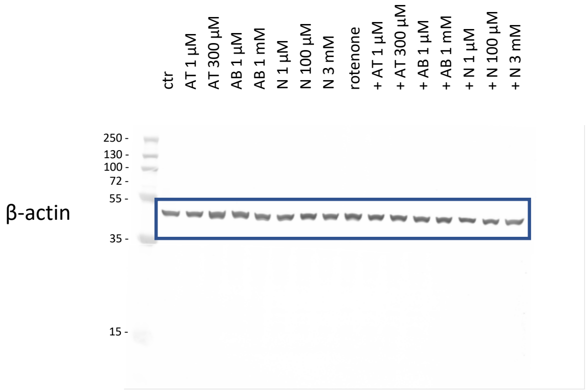

Full unedited blots for figure S4

Channel 1 (800 nm)

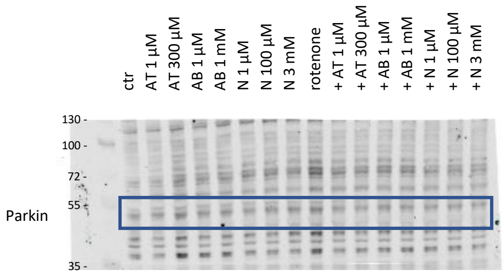

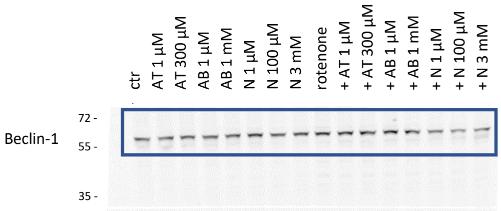

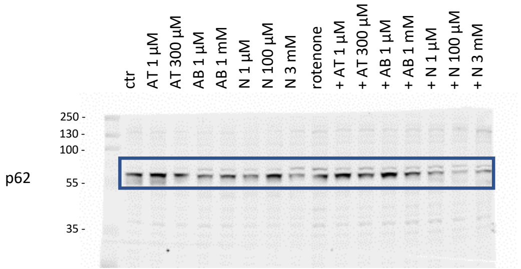

Channel 2 (680 nm)

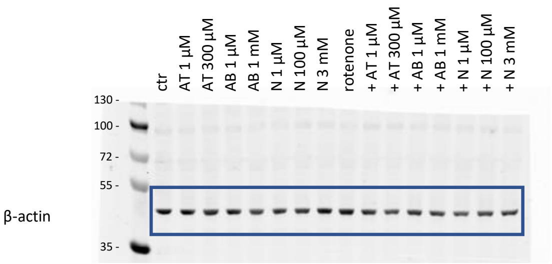

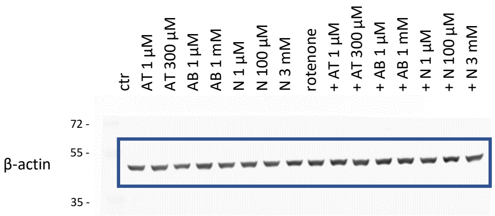

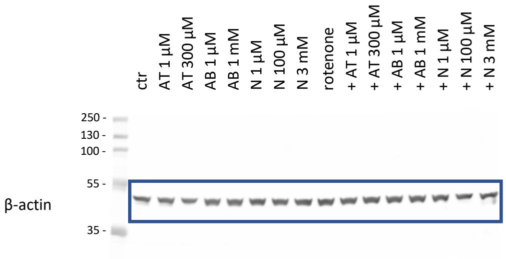

Supplement: Supplementary file 1 — Data S1: cns70571‐sup‐0001‐DataS1.pdf. [file CNS-31-e70571-s001.pdf]
