# Supplementary material for: Reorganization of Mitochondrial Function and Architecture in Response to Plant‐Derived Alkaloids: Anatabine, Anabasine, and Nicotine, Investigated in SH‐SY5Y Cells and in a Cellular Model of Parkinson's Disease
Source: CNS Neurosci Ther. 2025 Sep 4;31(9):e70571. doi: 10.1111/cns.70571 (PMC12409299; doi:10.1111/cns.70571)
Supplement: Supplementary file 2 — Data S2: cns70571‐sup‐0002‐DataS2.docx. Figure S1: (supplementary to Figure 1) Toxicity thresholds for the alkaloids anatabine, anabasine and nicotine in SH‐SY5Y cells. (a) Representative brightfield images of SH‐SY5Y cells treated for 1, 2, or 3 days with different concentrations of the tested alkaloids. The scale bar indicates 50 μm. (b) The results of cell viability determination with the MTT assay after 1, 2, and 3 days of treatment with different concentrations of the tested compounds. The data points present averages for Day 1 (diamonds), Day 2 (squares) and Day 3 (triangles) from 2 to 4 independent repetitions. (c) The impact of the tested compounds on cell counts after 3 days of treatment. The bar graphs present the means ± SDs from n = 3–4 independent repetitions. *p < 0.05 for Student's t‐test vs. untreated cells. Figure S2: (supplementary to Figures 2 and 5) Impact of anatabine, anabasine and nicotine on the morphology of SHSY‐5Y cells. Representative confocal images of SH‐SY5Y cells treated for 2 days with the investigated alkaloids (a) alone or (b) in presence of 50 nM rotenone. The cell nuclei (blue) were stained with DAPI, the actin cytoskeleton (red) was stained with phalloidin‐Alexa 546, and the microtubules (green) were visualized with an anti‐α‐tubulin antibody. The scale bars indicate 10 μm. AB, anabasine; AT, anatabine; N, nicotine. Figure S3: (supplementary to Figure 3) Analysis of the impact of anatabine, anabasine and nicotine on mitochondrial structure and function. (a) Representative measurement of oxygen consumption in SH‐SY5Y cells. (b) Impact of 2 days of treatment with the tested alkaloids on oxygen consumption in the presence of 1 μg/mL oligomycin and (c) maximal respiration obtained during CCCP titration. The graphs present the means ± SDs from n = 4 independent repetitions. *p < 0.05 for Student's t‐test vs. untreated cells. (d) Schematic representation of the image analysis workflow (detailed description in the Materials and Methods [file CNS-31-e70571-s002.docx]

**DATA S2**

**
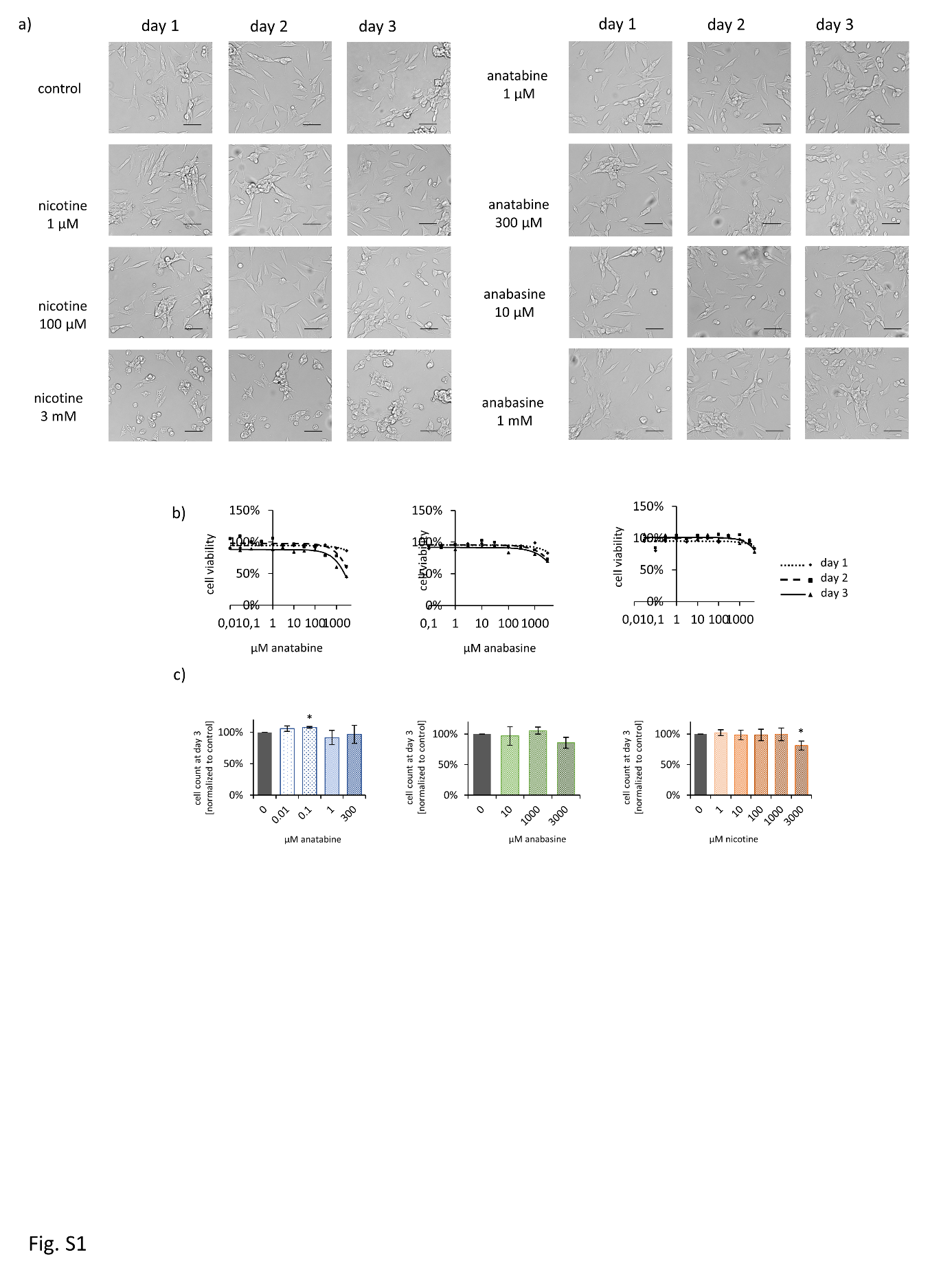
**

**Fig. S1 (supplementary to Fig. 1) Toxicity thresholds for the alkaloids anatabine, anabasine and nicotine in SH-SY5Y cells. a)** Representative brightfield images of SH-SY5Y cells treated for 1, 2 or 3 days with different concentrations of the tested alkaloids. The scale bar indicates 50 µm. **b)** The results of cell viability determination with the MTT assay after 1, 2 and 3 days of treatment with different concentrations of the tested compounds. The data points present averages for day 1 (diamonds), day 2 (squares) and day 3 (triangles) from 2--4 independent repetitions. **c)** The impact of the tested compounds on cell counts after 3 days of treatment. The bar graphs present the means ± SDs from n = 3–4 independent repetitions. * p < 0.05 for Student’s t-test vs untreated cells.


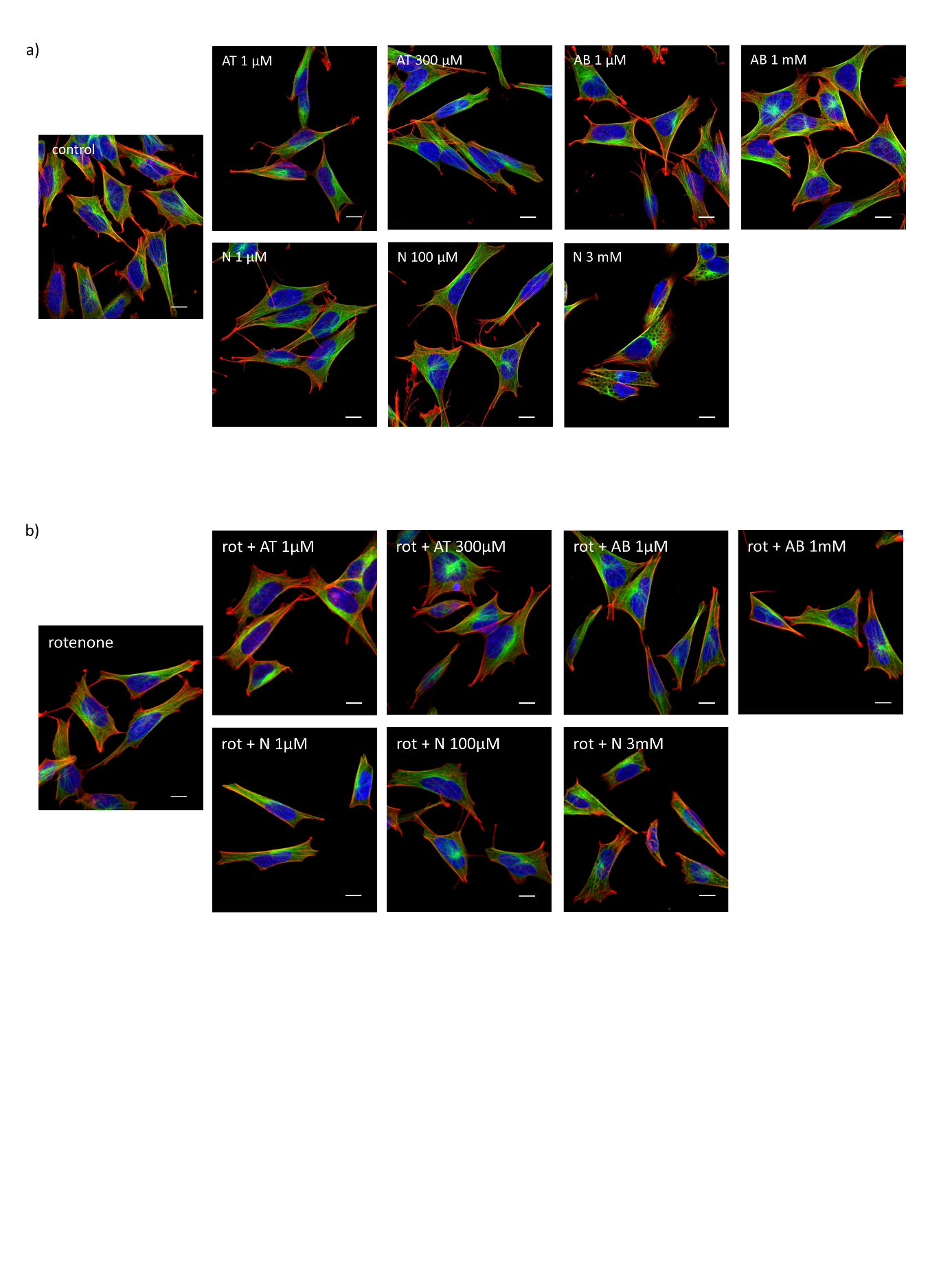


**Fig. S2 (supplementary to Fig. 2 and 5) Impact of anatabine, anabasine and nicotine on the morphology of SHSY-5Y cells**. Representative confocal images of SH-SY5Y cells treated for 2 days with the investigated alkaloids **a)** alone or **b)** in presence of 50 nM rotenone. The cell nuclei (blue) were stained with DAPI, the actin cytoskeleton (red) was stained with phalloidin-Alexa 546, and the microtubules (green) were visualized with an anti-α-tubulin antibody. The scale bars indicate 10 µm. AT – anatabine, AB – anabasine, N – nicotine

**
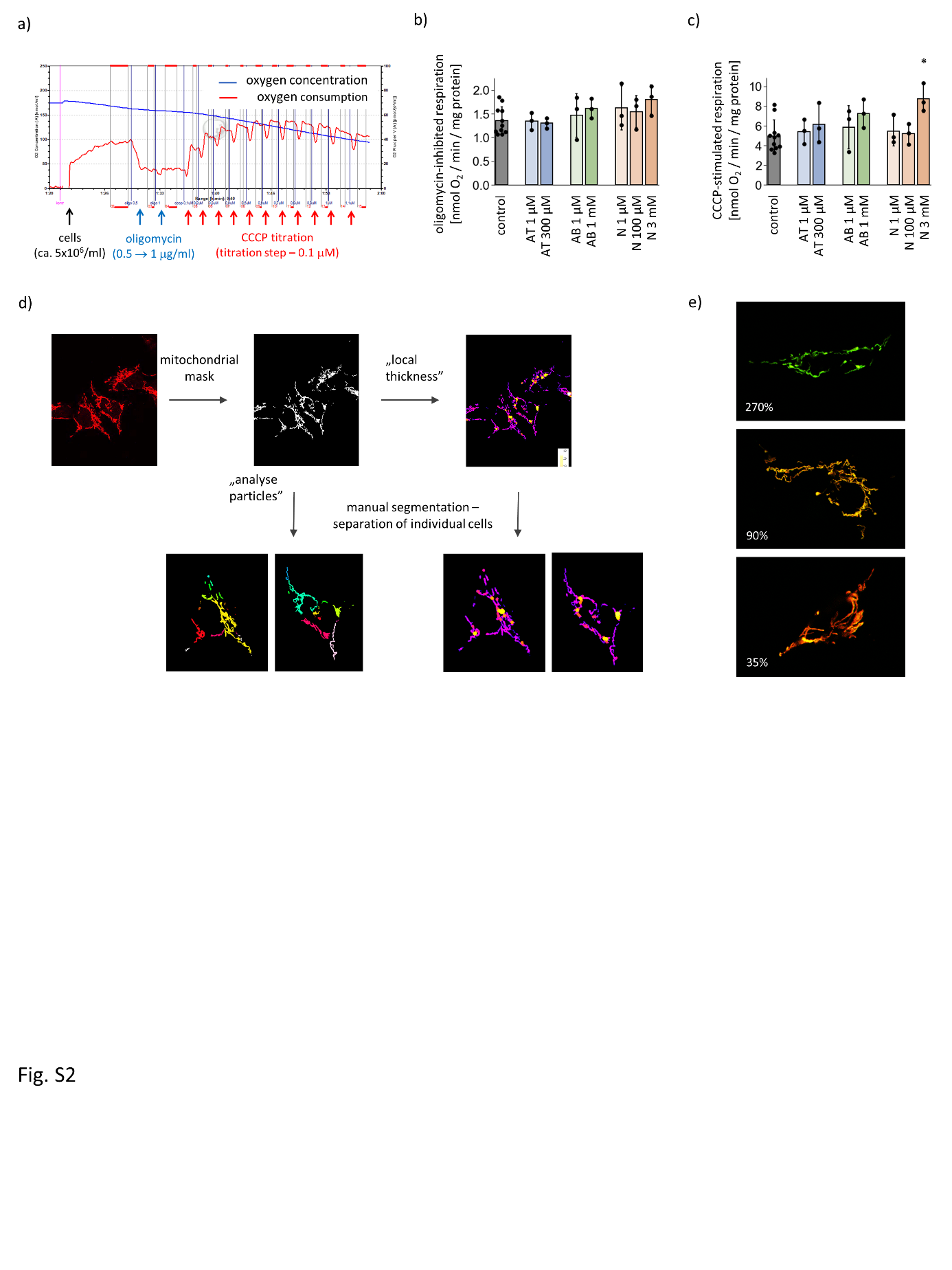
**

**Fig. S3 (supplementary to Fig. 3) Analysis of the impact of anatabine, anabasine and nicotine on mitochondrial structure and function. a)** Representative measurement of oxygen consumption in SH-SY5Y cells. **b)** Impact of 2 days of treatment with the tested alkaloids on oxygen consumption in the presence of 1 µg/ml oligomycin and **c)** maximal respiration obtained during CCCP titration. The graphs present the means ± SDs from n = 4 independent repetitions. * p < 0.05 for Student’s t-test vs untreated cells. **d)** Schematic representation of the image analysis workflow (detailed description in the Materials and Methods). **e)** Overlaid images of green and red fluorescence in SH-SY5Y cells transfected with the MitoTimer vector. Examples of cells with green/red fluorescence ratios corresponding to 270%, 90% and 35% of the average ratio measured in untreated cells.

**
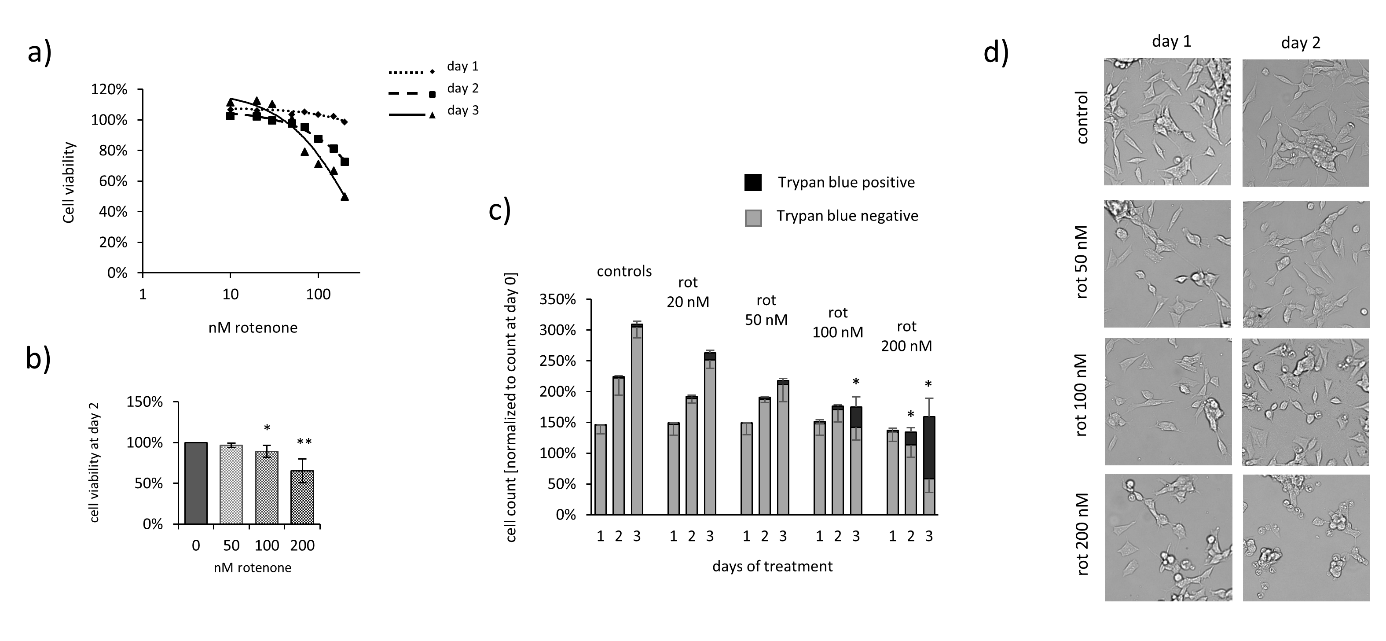
Fig. S4 Determination of the rotenone concentration causing mild cellular stress in SH-SY5Y cells** **a)** The results of cell viability determination with the MTT assay after 1, 2 and 3 days of treatment with different rotenone concentrations. The data points present averages for day 1 (diamonds), day 2 (squares) and day 3 (triangles) from 2--4 independent repetitions. **b)** Cell viability was measured with an MTT assay after 2 days of treatment with selected rotenone concentrations. **c)** Cell counts after 1, 2 and 3 days of treatment with selected rotenone concentrations. The results were normalized to the number of cells per well on the day of starting the treatment (day 0). The black parts of the bars represent the number of trypan blue-positive cells. **d)** Representative images of SH-SY5Y cells treated with selected rotenone concentrations for 1 and 2 days. The bar graphs in panel B present the means ± SDs from n = 6 and those in panel C from n = 3 independent experiments. ^*^ p < 0.05, ^**^ p < 0.01 according to Student’s t-test vs untreated cells.

**
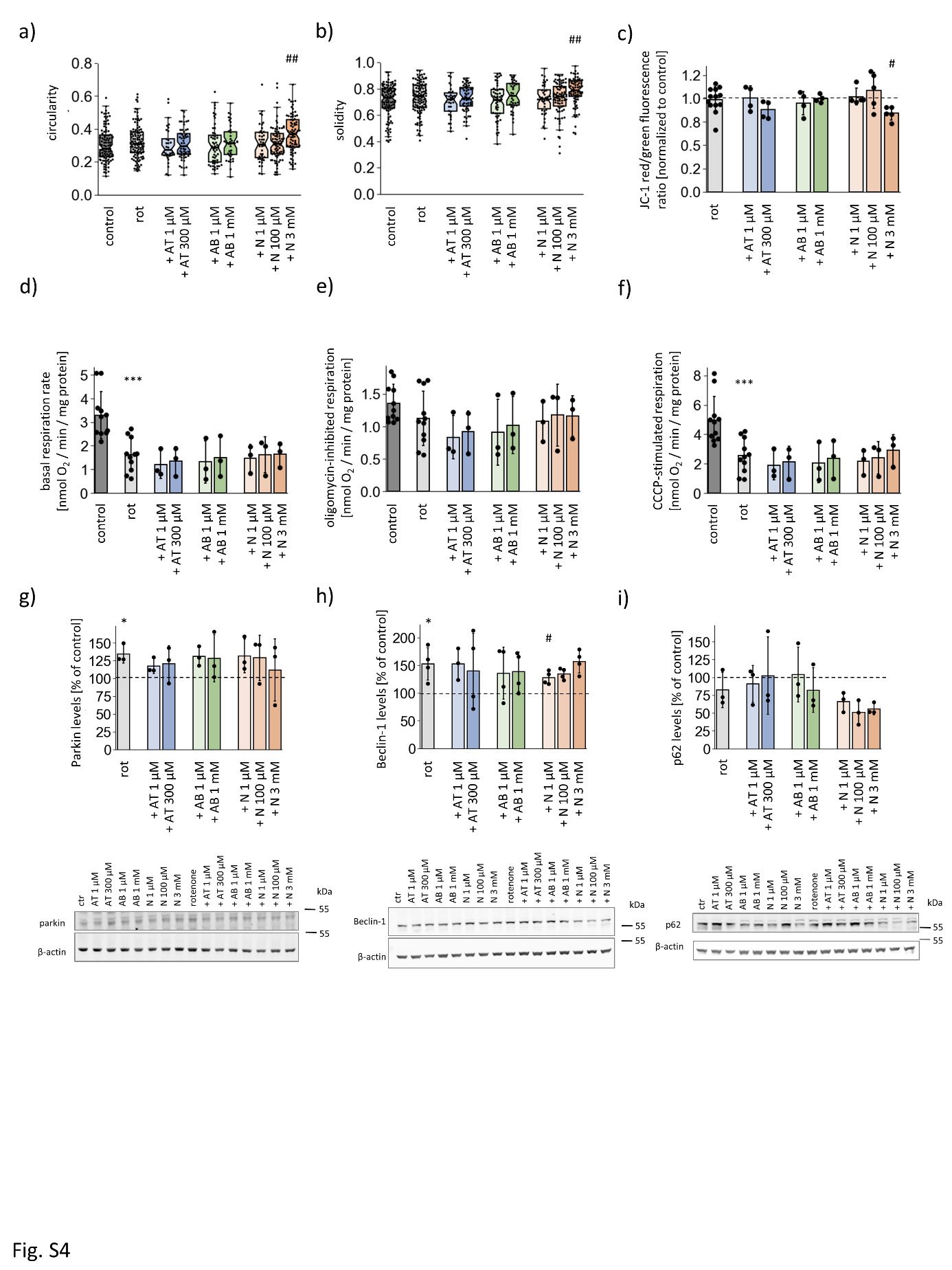
Fig. S5 (supplementary figures 5 and 6)** **Cellular morphology (a, b), mitochondrial function (c-f) and the levels of auto/mitophagy effectors (g-i) in SH-SY5Y cells treated for 2 days with rotenone alone or in combination with the investigated alkaloids** Dashed lines mark the average values measured in untreated cells. **a)** Cell shape circularity, **b)** cell shape solidity, **c)** mitochondrial membrane potential, **d)** basal respiration rates, **e)** oligomycin-inhibited respiration, **f)** cccp-uncoupled respiration, **g)** the levels of parkin, **h)** beclin-1 and **i)** p62 proteins. Representative Western blots are shown below the graphs. The graphs present the means ± SDs from the analysis of n = 34 - 116 individual cells (a, b), n = 4 independent experimental repetitions or n = 3-4 independent lysate sets. * p < 0.05, *** p < 0.001 for Student’s t-test vs untreated cells; ^#^ p < 0.05, ^##^ p < 0.01 for Student’s t-test vs rotenone-treated cells. AT – anatabine, AB – anabasine, N – nicotine.
